# Perineal Membrane Structural Changes: An Unstudied Aspect of Prolapse

**DOI:** 10.1007/s00192-026-06614-7

**Published:** 2026-05-02

**Authors:** Fernanda Pipitone, Carolyn W. Swenson, John O. L. DeLancey, James A. Ashton-Miller, Luyun Chen

**Affiliations:** 1 Disciplina de Ginecologia, Departamento de Obstetricia e Ginecologia, Faculdade de Medicina, Universidade de Sao Paulo, Av Dr Eneas de Carvalho Aguiar, 255, Sao Paulo, 05403-000 Brazil; 2 Pelvic Floor Research Group, University of Michigan, 1500 E. Medical Center Dr., Ann Arbor, MI 48109 USA; 3 Department of Obstetrics and Gynecology, University of Utah, 324 10Th Ave #200, Salt Lake City, UT 84103 USA; 4 Department of Obstetrics and Gynecology, University of Michigan, 1500 E. Medical Center Dr., Ann Arbor, MI 48109 USA; 5 Department of Mechanical Engineering, University of Michigan, 2350 Hayward, Ann Arbor, MI 48109 USA; 6 Department of Biomedical Engineering, University of Michigan, 2350 Hayward, Ann Arbor, MI 48109 USA

**Keywords:** Magnetic resonance imaging, Pelvic organ prolapse, Perineal membrane, Urogenital diaphragm, Urogenital hiatus

## Abstract

**Introduction and Hypothesis:**

The perineal membrane (PM), perineal body, and levator ani muscles form the perineal complex, which is responsible for hiatal closure. Yet failures in its connective tissues that may lead to hiatal closure impairment are poorly understood. We tested the hypothesis that pelvic organ prolapse involves PM abnormalities by comparing PM morphology between young women with prolapse and parous controls using a validated MRI-based reconstruction and analysis technique.

**Methods:**

This is a secondary analysis of MRIs from two prior studies. The PM was traced on coronal scans in 3D Slicer^®^, and surface models were analyzed using Rhino^®^. Six PM parameters were measured: swinging door angle, visible bony origin length, hiatal anteroposterior diameter and area, PM surface area, and midline separation. Group comparisons used *t* tests, with Cohen’s *d*, correlations, and stepwise regression analysis.

**Results:**

Resting MRIs from 17 young parous women (aged < 40 years) with prolapse and 20 parous controls were compared. Women with prolapse showed 23% greater midline separation of the PM, 30% larger hiatal area, 26% larger hiatal anteroposterior diameter, 17% longer bony origin, and 26% larger PM surface area (all *p* ≤ 0.002, *d* = 1.1–1.6); swinging door angle was similar between groups (*p* = 0.60). Levator ani status, midline separation, and PM surface area independently predicted prolapse, explaining 76% of variance.

**Conclusions:**

The PM structure was altered in those with prolapse through loss of central PM connection, hiatal elongation, and hiatal widening. Larger studies are needed to confirm and guide targeted repair techniques that could potentially prevent prolapse development and/or progression.

## Introduction

The pelvic organ support system comprises several elements. In levels I and II, the pelvic organs are attached to the pelvic walls by connective tissues. In level III, support is provided by the perineal complex, which consists of the perineal membranes (PM), perineal body, the medial levator ani (LA) muscles, and the endopelvic fascia, which unites these elements. The two sides of the PM are centrally connected through a portion of the perineal body and together form the connective tissue component of the perineal complex (PC_CT_) [1], a region that may be of particular importance for maintaining hiatal closure as it bridges the two sides of the pelvic floor [2]. Yet comparative data between women with and those without pelvic floor dysfunction to test hypotheses regarding PC_CT_ structural failure and overall PM morphological changes are limited. This is a major deficiency in our understanding of the hiatal closure mechanism and therefore of how hiatal enlargement plays a role in the development of prolapse.

The PM is a three-dimensional network of tissue deep in the perineal space that takes origin from the ventral two thirds of the ischiopubic rami bilaterally, and spans medially to insert into the periurethral tissues, vaginal wall, and perineal body. Histological studies demonstrate that the LA muscles not only bound the perineal membrane cranially but their fibers insert directly into the PM connective tissue bands [3–5], suggesting a structural arrangement able to carry tensile loads. Also, ventrally, the LA fascia is continuous with the PM, again signaling a tensile load-carrying pathway. The left and right PMs then insert dorsally on either side of the perineal body, thereby connecting the two halves of the pelvis. This interconnected network of structures forms the perineal complex and is a key aspect of level III pelvic support.

The lack of understanding of the role of the PM in hiatal closure is largely attributable to its deep location within the perineum, which makes it not readily visible on clinical examination and poses significant challenges for direct assessment. Fortunately, with the advent of advanced soft-tissue imaging—in particular thin-slice MRI scans and 3D endovaginal ultrasound—research efforts are being made to better understand PM changes and the role of PC_CT_ in pelvic support [6, 7].

With this knowledge gap in mind, we sought to test the hypothesis that PM and PC_CT_ alterations are involved in the prolapse mechanism in existing cohorts of young women with and without prolapse by comparing their PM morphology using a previously published MRI-based reconstruction technique [6].

## Materials and Methods

This retrospective case–control study was based on a secondary analysis of a convenience sample of pelvic MRIs obtained from two prior IRB-approved studies of vaginally parous women (HUM 00132937; HUM 00051193). All included participants underwent the same thin-slice (2 mm) pelvic floor MRI protocol, which was required for the present morphological analysis [8, 9]. Cases were selected from the first parent study, which enrolled parous women younger than 40 years old with symptomatic pelvic organ prolapse confirmed on physical examination (lowest POP-Q points ≥  + 1). Controls were drawn from the second study (Evaluating Maternal Recovery from Labor and Delivery [EMRLD]), in which primiparous women underwent pelvic MRI 8 months postpartum. For this second parent study, participants had to meet at least one of the following risk factors for pelvic floor injury: length of second stage > 150 or < 30 min, forceps-assisted delivery, maternal age > 35 years, and newborn birth weight > 4000 g. Despite the presence of these risk factors, controls were required to have normal pelvic support on physical examination, defined as no POP-Q points beyond the hymen (Ba, Bp, and C < 0). Participants with a history of obstetric anal sphincter injury were excluded, as such injury could alter the perineal anatomy and limit visualization and morphological assessment of the PM.

The MRI protocols are described in detail in previous publications [8, 9]. In brief, women underwent MRI in the supine position, and 2-mm proton density-weighted sequences were performed in the axial, coronal, and sagittal planes (3 T Philips Achieva, Philips Medical System, Eindhoven, The Netherlands).

**Fig. 1:**
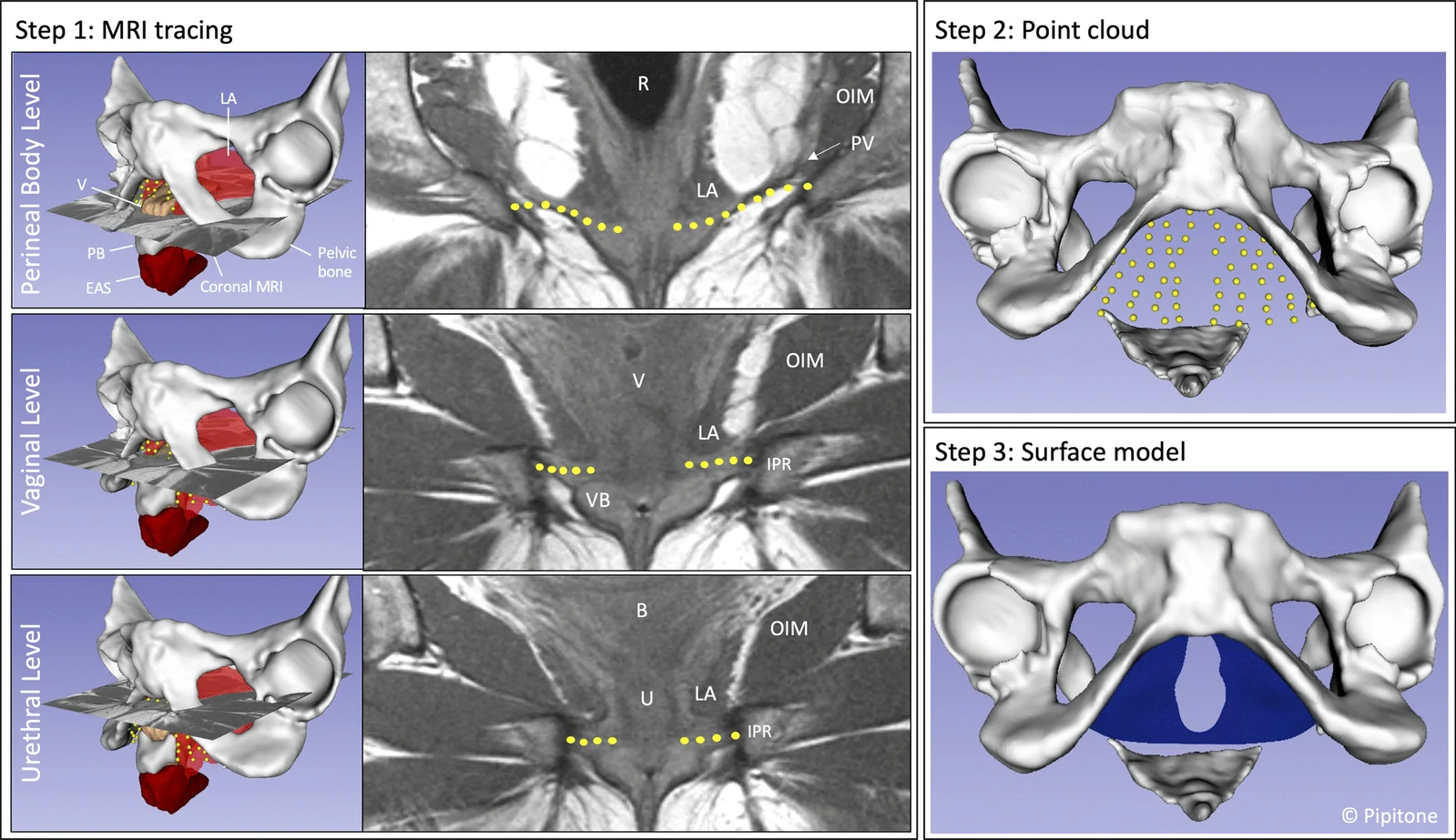
Perineal membrane reconstruction method. Step 1: left column shows left oblique view of the pelvis with a perineal membrane array of fiducials (yellow dots) transected by a coronal MRI scan at three different anatomical levels as labeled (rows); right column shows corresponding coronal MRI scans with perineal membrane tracing (yellow dots). Step 2: caudal view of the pelvis with the resulting array of fiducials (yellow dots). Step 3: caudal view of the pelvis with the final perineal membrane surface model. Modified from Pipitone et al. [6]. *EAS* external anal sphincter, *LA* levator ani muscle, *PB* perineal body, *V* vagina, *B* bladder, *IPR* ischiopubic ramus, *OIM* obturator internus muscle, *PV* pudendal vessels, *R* rectum, *U* urethra, *VB* vestibular bulb

The PM reconstruction technique was conducted as previously published (Fig. 1) [6]. In step 1, the PM was traced on coronal MRI scans (slice thickness 2 mm, TE 261, TR 1800) for each subject using 3D Slicer^®^ software (v 4.5.0, www.slicer.org, Brigham and Women’s Hospital, Boston, MA, USA); anatomical landmarks were determined according to previous histological and imaging studies [3, 10]. Medial and lateral borders of fiducial placement were confirmed on the axial plane. In step 2, an array of fiducials covering the entire extent of the PM was placed. Ventrally, the PM was marked until corresponding fiducials on the sagittal plane reached the infero-posterior margin of the arcuate pubic ligament. Dorsally, fiducials were placed until the last coronal slice, where lateral origins on the ischiopubic rami could be identified, corresponding to the level of the perineal body. Care was taken not to include the pudendal vessels that enter the PM at this location. In step 3, this resulting array of fiducials was imported into Rhino® software (V 6.0 SR23, Robert McNeel & Assoc.) to build a surface model, based on which measurements were made and quantitative analysis was carried out.

**Fig. 2:**
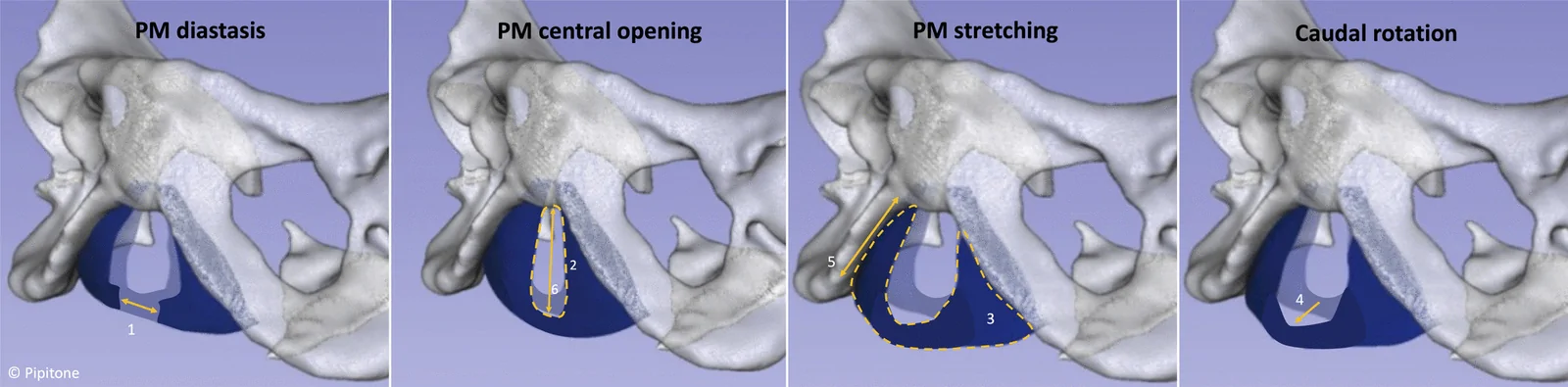
Perineal membrane (PM) structural changes and their corresponding morphological parameters (yellow arrows and dashed areas). The numbers in each panel correspond to the morphological parameters listed in the text and also shown in Fig. 3. Modified from Pipitone et al. [6]

To compare the morphology of PMs of the two groups, the following six parameters were measured [6]: midline separation (1), hiatal area (2), PM surface area (3), swinging door angle (4), visible length of the bony origin (5), and hiatal anteroposterior (AP) diameter (6). All parameters were normalized for bony pelvis size except for swinging door angle, as the latter is, by definition, not influenced by pelvic bone dimension but rather reflects a caudal rotation of the PMs. These parameters were based on prior anatomical and imaging studies [3, 10–13], to reflect PM structural changes and PC_CT_ alteration that could be potentially associated with pelvic organ prolapse (Fig. 2). Of note, the hiatal area measured in this study corresponds to the central opening of the PM, which represents the hiatus at a slightly more caudal level than where it is often measured on imaging studies [14].

The LA muscles were scored on MRI scans for all subjects according to a published method [15], yielding a total score from 0 to 6. This variable was divided into none (0) or minor defect (1–3) versus major defect (≥ 4 or unilateral 3), as previous studies have shown that lesser defects are not associated with prolapse [16].

Student’s *t* tests were used to compare the PM parameters of the two groups and percentages of mean difference. Given the exploratory nature of the analyses and the limited sample size, no correction for multiple comparisons was applied, as such adjustments would substantially reduce statistical power; instead, effect sizes were reported to aid interpretation of the magnitude and clinical relevance of observed differences. Cohen’s *d* effect sizes were categorized as large (≥ 0.80), medium (≥ 0.50), and small (≥ 0.20) [17]. Pearson correlation coefficients were calculated to examine linear associations among LA status, midline separation, PM surface area, visible bony origin length, hiatal area and AP diameter, and swinging door angle. The resulting correlation matrix was visualized as a color-coded heatmap, with red indicating positive and blue indicating negative correlations. Statistical significance was set at *p* < 0.05. A stepwise multiple linear regression analysis with forced order entry was performed to identify the relative contribution of midline separation, LA status (major vs no major defect), and PM surface area to hiatal area. Predictors were entered sequentially in a predetermined order based on hypothesized anatomical relevance. At each step, model fit was evaluated using *R*^*2*^, adjusted *R*^*2*^, *F* statistics, and corresponding significance levels. Standardized coefficients (*B*) were also reported for each predictor in the final model. All statistical analyses were performed using SPSS (version 27; IBM, Armonk, NY, USA).

## Results

A total of 17 young women with prolapse (yPOP) and 20 parous controls without a history of anal sphincter laceration had available MRI scans with adequate coverage of PM topography for the present analysis. The total cohort had an average age and BMI of 33.0 ± 5.5 years and 25.8 ± 5.1 kg/m^2^ respectively. Women in the yPOP group were on average 6.5 years older than those in the control group, had an average parity of 3.1 ± 1.8 and an average maximum prolapse (lowest POP-Q point) of + 1.3 ± 1.0 cm. In addition, the rate of major LA defects was twice as high in the prolapse group than in the controls (20% vs 41%, *p* = 0.160) and similar to the defect rate in prolapse cohorts reported in the literature [16]. Groups were similar regarding BMI. Clinical-demographic data are shown in Table 1.

**Table 1:** Clinical-demographic data by group

	Parous controls, *n* = 20	Young prolapse, *n* = 17	*p* value
Age, years	30.0 (4.8)	36.5 (4.1)	< 0.001
BMI, kg/m^2^	25.1 (4.2)	26.7 (5.8)	0.344
Ethnicity - *n* (%)			0.044
White	18 (1)	14 (1)	
Black	0 (0)	3 (0)	
Other	2 (0)	0 (0)	
Parity	3.1 (0.0)	1.0 (0.1)	
Maximum prolapse, cm	−2.4 (0.7)	1.3 (1.0)	< 0.001
POP-Q value, cm			
Ba	−2.6 (0.5)	0.4 (1.5)	< 0.001
Bp	−2.5 (0.7)	−0.2 (1.2)	< 0.001
C	−8.0 (0.2)	−3.3 (3.6)	< 0.001
GH	2.9 (1.1)	4.9 (1.0)	< 0.001
PB	2.1 (0.6)	3.6 (0.9)	< 0.001
TVL	12.2 (0.8)	9.7 (1.5)	< 0.001

Data are presented as mean (SD) unless otherwise indicated

*BMI* body mass index, *POP-Q* Prolapse Organ Prolapse Quantification

**Fig. 3:**
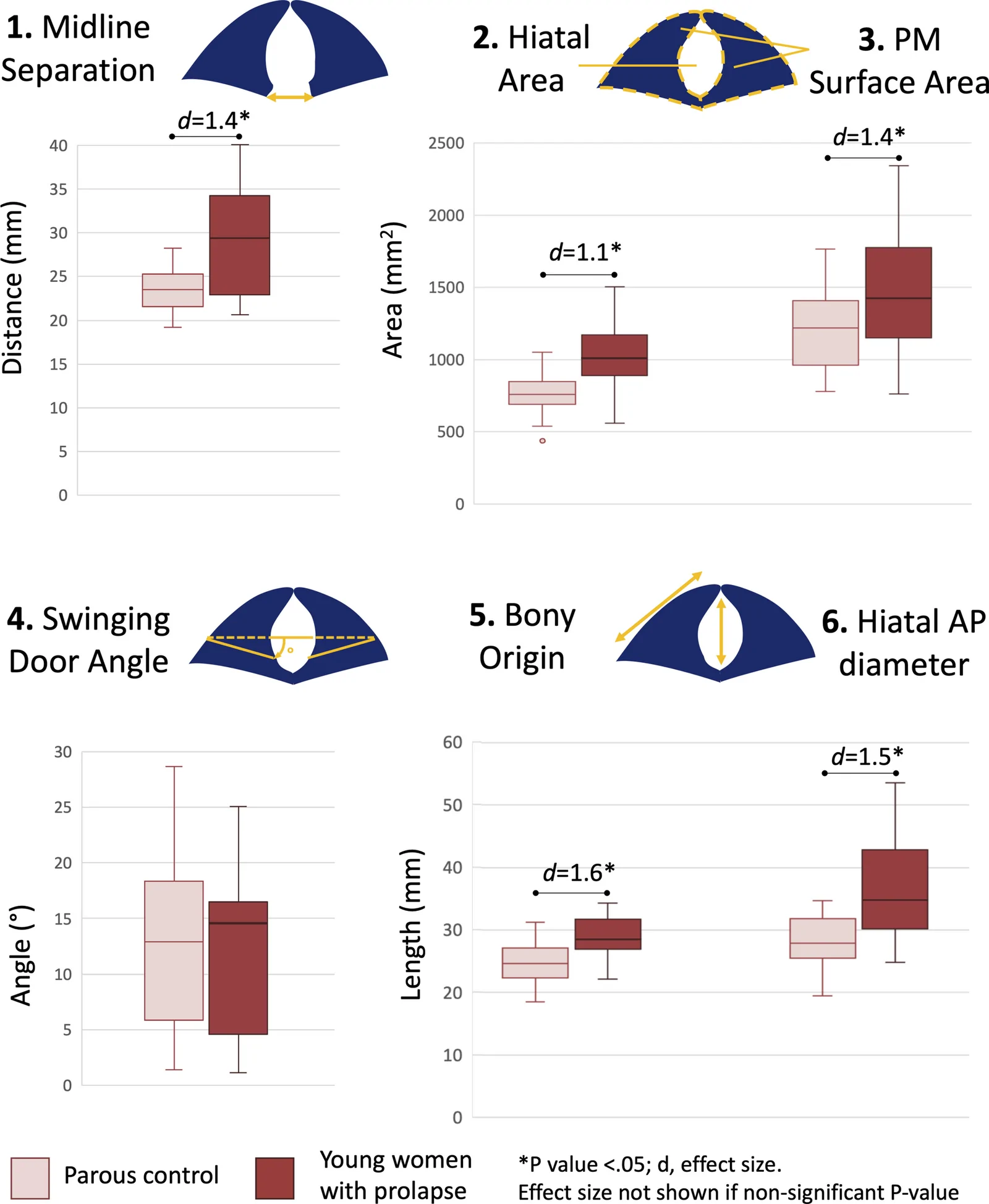
Perineal membrane morphological parameters. Group comparison for each perineal membrane morphological parameter (1–5), as labeled at the top of the graphs. The blue icons represent the perineal membrane surface model with corresponding measurements illustrated in yellow. *p < 0.05; *d* is Cohen’s effect size. Effect size is not shown if the *p* value is nonsignificant

In the PM morphological analysis, there were statistically significant differences in all parameters between the groups except for the swinging door angle (Fig. 3). Women with prolapse had 23% larger midline separation than controls (*p* < 0.001, *d* = 1.4). The yPOP group also showed greater dimensions of the PM central opening: women in this group had a 30% larger PM hiatal area (*p* < 0.001, *d* = 1.4) and a 26% larger hiatal AP diameter (*p* < 0.001, *d* = 1.6) than parous controls. In addition, an overall stretching of the PM was seen: a 17% longer visible bony origin length (*p* < 0.001, *d* = 1.5) and a 26% larger surface area (*p* = 0.002, *d* = 1.1) in yPOP women. Groups had similar average swinging door angles (*p* = 0.60, *d* = 0.2).

**Fig. 4:**
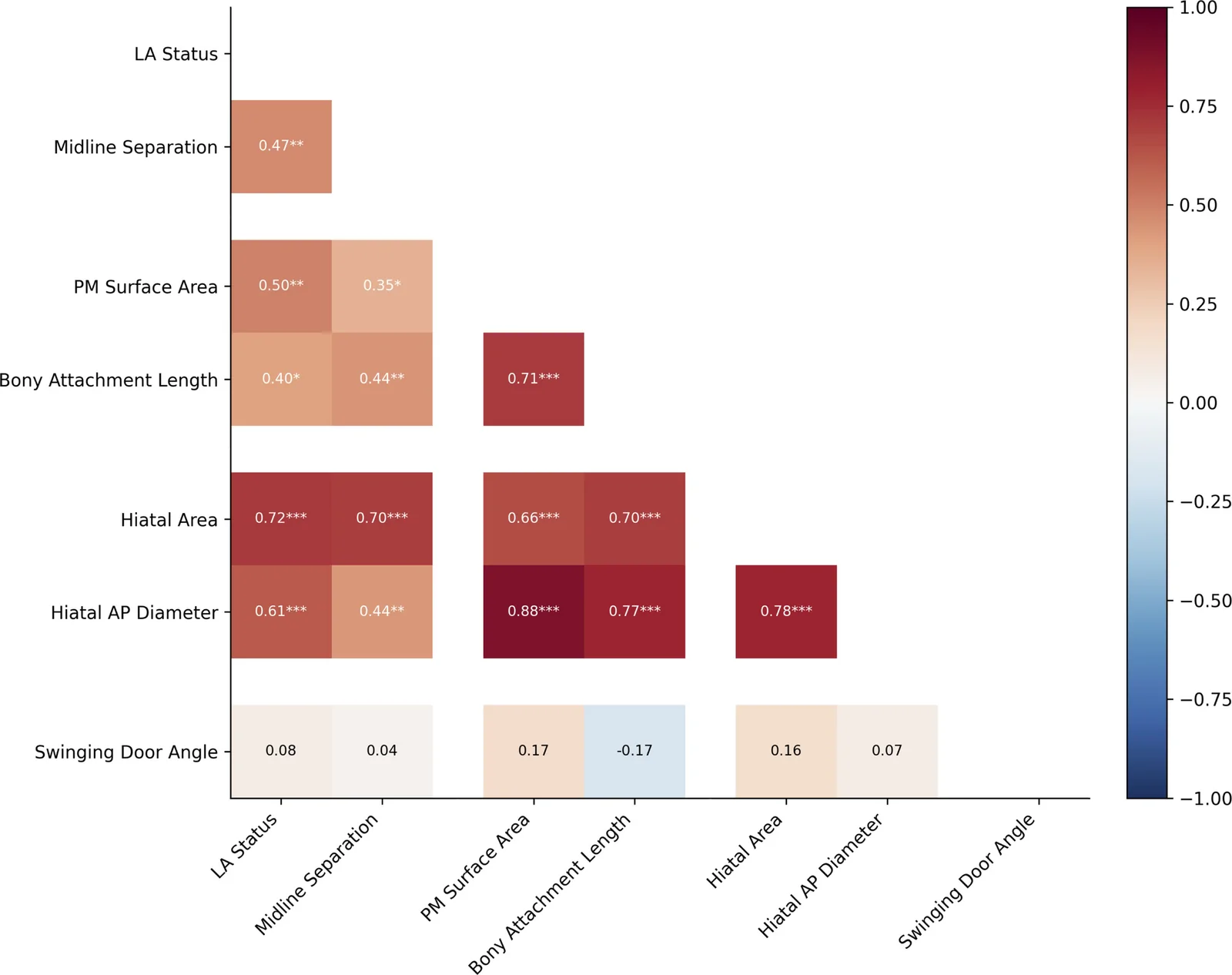
Correlation matrix showing associations among clinical, demographic, and imaging variables. Cells are color-coded according to the strength and direction of Pearson correlation coefficients (red = positive, blue = negative). Darker colors indicate stronger correlations. Asterisks denote statistical significance (*p < 0.05, **p < 0.01, ***p < 0.001). *LA* levator ani, *PM* perineal membrane

Pearson correlation analysis was performed to explore associations among clinical and imaging variables (Fig. 4). Significant positive correlations were found between LA status and midline separation (*r* = 0.47, *p* < 0.01), PM surface area (*r* = 0.50, *p* < 0.01), hiatal area (*r* = 0.72, *p* < 0.001), and hiatal AP diameter (*r* = 0.61, *p* < 0.001). Similarly, midline separation correlated strongly with hiatal area (*r* = 0.70, *p* < 0.001) and hiatal AP diameter (*r* = 0.44, *p* < 0.01). As expected, measures reflecting PM central opening—hiatal area and hiatal AP diameter—were highly intercorrelated (*r* = 0.78, *p* < 0.001), as were PM surface area and visible bony origin length (*r* = 0.71, *p* < 0.001). In contrast, swinging door angle showed no meaningful correlation with any of the other parameters (*r* ≤ 0.17, *p* > 0.05).

A mechanistically informed stepwise regression analysis was performed to assess the contribution of midline separation, LA status, and PM surface area to the hiatal area size (Table 2). In the first step, midline separation explained 49% of the variance in hiatal area (*R*^*2*^ = 0.49, adjusted *R*^*2*^ = 0.47, *p* < 0.001). Adding LA status increased the explained variance to 68% (*R*^*2*^ = 0.68, adjusted *R*^*2*^ = 0.66, *p* < 0.001). When PM surface area was entered in the final step, the model accounted for 76% of the total variance (*R*^*2*^ = 0.76, adjusted *R*^*2*^ = 0.74, *p* < 0.001). In the final model, all three variables remained significant independent predictors of hiatal area: LA status (*B* = 38.0, *p* = 0.002), midline separation (*B* = 17.9, *p* < 0.001), and PM surface area (*B* = 0.20, *p* = 0.002).

**Table 2:** Stepwise regression model to analyze factors determining the hiatal area

Predictors	*R*^*2*^	Adjusted *R*^*2*^	*F*	*B*	*p* value
Step 1 model:					
Midline separation	0.49	0.47	33.07	17.91	< 0.001
Step 2 model:					
Midline separation + LA status	0.68	0.66	35.71	37.97	< 0.001
Step 3 model:					
Midline separation + LA status + PM surface area	0.76	0.74	34.91	0.20	< 0.001

*LA* levator ani, *PM* perineal membrane

## Discussion

### Principal Findings

The morphology of PMs differed between young women with prolapse and women with normal support, demonstrating an association between this pelvic floor disorder and PM structural changes. Midline separation was 23% larger in the young women with prolapse and may represent a diastasis of the pelvic floor much like a diastasis rectus abdominis, in which the two rectus abdominis muscles separate along the midline of the abdominal wall; this PC_CT_ alteration might potentially impair hiatal closure. In addition, hiatal area and AP diameter were 30% and 26% larger respectively in women with prolapse.

It is noteworthy that the observed morphological changes—loss of central connection, hiatal elongation and widening—were associated with prolapse in a cohort of young women, that is, before notable age-related changes to the pelvic floor occur. The present study design does not allow for causal inference; however, it prompts consideration of whether these PC_CT_ alterations precede the development of pelvic organ prolapse and might be involved as a causal factor in the disease pathway, or whether they represent tissue remodeling consequent to the presence of prolapse. Further research will be needed to answer these questions.

Swinging door angle was the only PM morphological parameter similar between groups. This finding was expected, as previous preliminary data have shown differences in this parameter between nulliparous and parous women, but not between modes of delivery [6]. Rather than reflecting a birth-related change or injury, this morphological feature may represent a physiological adaptation of pregnancy in preparation for childbirth.

### Interpretation and Implications

Pelvic organ prolapse arises from a complex interplay of genetic predisposition, reproductive events, aging, and lifestyle exposures that act on the anatomically complex pelvic support system over the lifespan [18]. Given this multifactorial etiology, no single alteration is likely responsible for hiatal failure. Our findings advance current knowledge by identifying potential prolapse-associated PM morphological changes and PC_CT_ alterations, which are a critical yet understudied component of pelvic organ support.

The perineal complex functions as a system, and as such, its several interdependent components cannot be addressed as separate structures but rather as an integrated network that operates as a functional unit to maintain hiatal closure. Thus, analogous to other systems, failure of one aspect of the complex can be compensated for by others. For example, loss of the midline connection between the sides of the PM may be compensated for by well-functioning LA muscles, as seen in women with a chronic anal sphincter defect with near-complete loss of the perineal body but a small genital hiatus on examination [19]. However, de-compensation can take place (i.e., the hiatus enlarges), when a critical combination of elements fails at the same time or when the compensatory capacity of the system is reduced by age or other intervening factors; thereafter, a previous injury to an element can no longer be compensated for by the others. This is well illustrated by our regression model, where midline separation alone explains 47% of hiatal area variation, whereas when in combination with LA defect, that percentage increases to 68%.

The interdependent nature of the perineal complex components has been previously described in cadaver studies [2, 12]. DeLancey performed anatomical dissection of 64 cadavers and demonstrated that when the distal rectum is subjected to increased force caudally, the PM on either side becomes taut and resists further displacement [2]. This ability of the PM to resist displacement relies on the structural continuity between its two sides through the perineal body—when transecting this connection, the author reported it then being possible for the distal rectum to be displaced downward. Our findings are also supported by Soga et al. who observed in a cadaver study that the two medial ends of the PM (referred to by them as the “lateral extensions of the perineal body”) tended to protrude caudally toward the subcutaneous layer in women with a history of vaginal delivery [12].

**Fig. 5:**
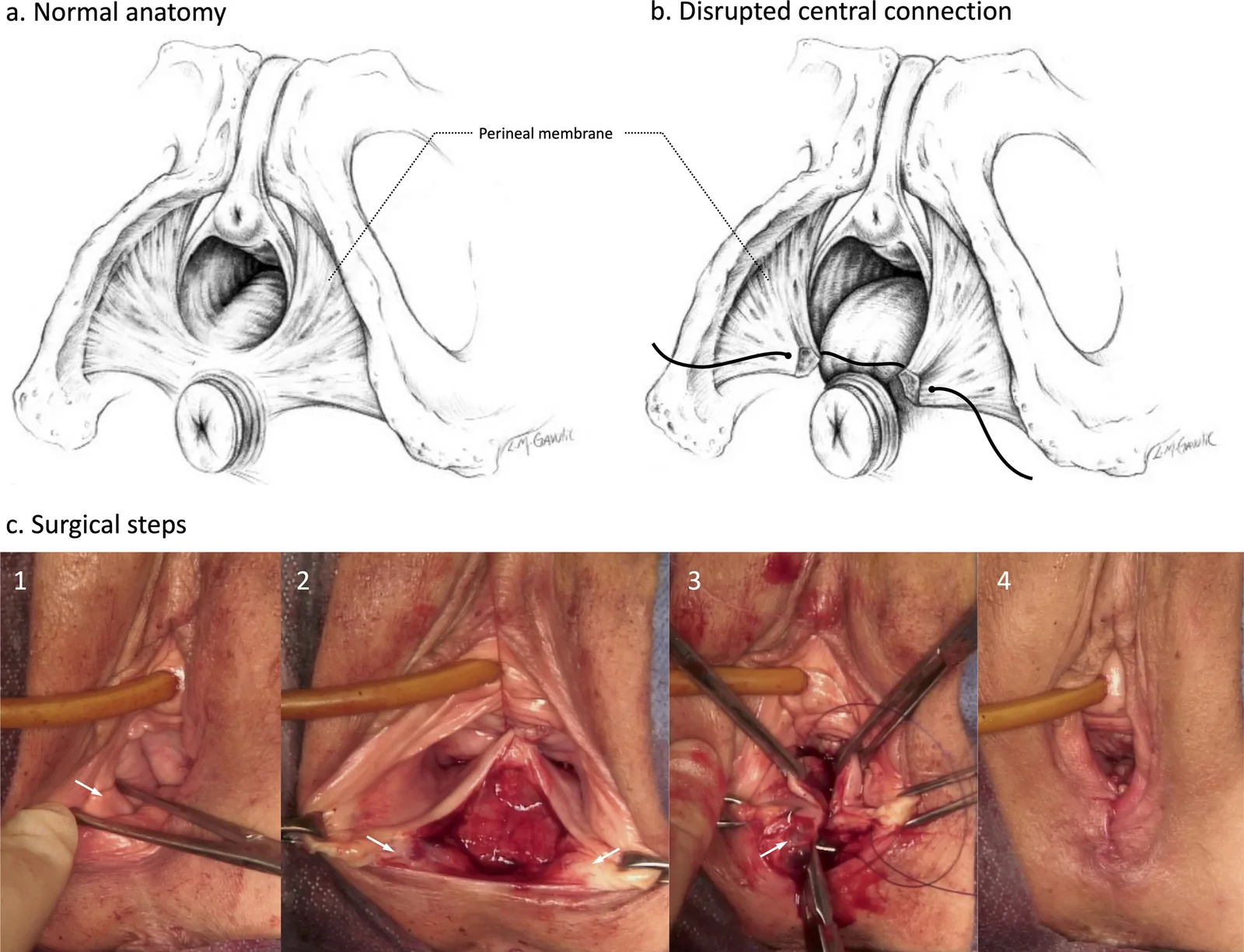
Illustration of the concept and surgical repair of central disruption of the perineal complex soft tissues, potentially associated with the development of prolapse. **a** Normal perineal complex anatomy. **b** Central disruption and a critical suture (solid black line) for the intended repair; note that the intervening vaginal tissue has not yet been excised, as it would be during surgery. **c** Key surgical steps (1–4) to reconnect the two medial ends of the perineal membrane (white arrows) to the midline: C1, deeply grasp the end of the perineal membrane just inside the hymen; C2, remove the intervening vaginal wall; C3, after closure of the upper portions, a targeted suture is placed to grasp the end of the membrane held in the clamp, equivalent to the suture illustrated in **b**; C4, completed repair

Studying PM morphological changes is clinically relevant because PC_CT_ alterations, especially the loss of their insertion on the perineal body, may be surgically repairable. The midline PM insertion lies immediately above the hymen, thus making it identifiable on pelvic examination and surgically accessible for central reconnection. In the experience of one of the senior authors (JOLD), this can be done in the operating room by grasping this tissue at the 4 and 8 o’clock positions at and just above the hymenal remnants with an Alis clamp (Fig. 5). Then, pulling toward the opposite side, one can confirm whether or not this bite contains the PM by palpating the area between the vagina and ischiopubic ramus to feel whether it has been made taut by this maneuver. As the LA muscles are intimately connected to the PM, bringing the separated PM and perineal body back to the midline when performing a posterior repair may plausibly re-align the LA and potentially place it more medially. This could help to restore the tensile load-carrying network that is formed by the interconnections between the LA, PM, and perineal body. We further hypothesize that this could be the phenomenon underlying the beneficial effect that surgically improving hiatus size has on surgical success rates [20]. This becomes especially relevant considering that clinical and surgical management of LA damage is currently limited [21].

Furthermore, it is possible that deeper obstetric perineal lacerations, frequently encountered during vaginal births, involve the perineal complex components and affect hiatal closure by loss of this central connection. This connection occurs at a specific location within the perineal body and does not involve all of it [22], and such injuries would remain subclinical during the early postpartum years. Currently, there is insufficient evidence to recommend surgical repair over nonsurgical management of second-degree perineal lacerations at the time of birth. Although some studies suggest that leaving such lacerations unsutured could reduce pain, analgesia use, and dyspareunia, these findings are based on short-term follow-up (6–12 weeks) [23] and do not address long-term perineal outcomes or pelvic floor function [21, 22]. Owing to this limited evidence, current guidelines advise that the decision to repair should be based on clinical judgment [24]. This judgment is typically guided by two factors: achieving hemostasis and restoring normal anatomy [25]. The second factor—proper anatomical alignment of all wound layers—remains greatly under-recognized, and many obstetricians and midwives are trained to regard the absence of bleeding as sufficient, overlooking the potential long-term consequences of suboptimal anatomical restoration.

Perineoplasty and other forms of posterior repairs are procedures commonly performed by gynecological surgeons; and yet, there is a lack of standardization of surgical technique and scientific-based criteria for choosing which patients would benefit [26]. If the involvement of PC_CT_ midline disruption in prolapse showing in the results are confirmed in a larger cohort study, clinical trials could be conducted to establish efficacy in reducing prolapse progression/development and best repair technique, and to identify optimal timing of the intervention for women whose anatomy was not repaired intrapartum.

Imaging modalities could play a valuable role in advancing our knowledge on the function and anatomy of the perineal complex. Assessment of this region represents a challenge for cadaver dissection or intraoperative evaluation as multiple distinct yet interconnected structures cross or attach to the perineal body making it difficult to distinguish one structure from another [27].

Although MRI can provide detailed imaging of pelvic floor structures, its cost and limited availability may restrict its use in larger studies and transfer to clinical practice. Ultrasound, however, enables close-proximity, real-time visualization of the perineal complex and can help to elucidate the discrete elements underlying what appears to be a uniform structure [28, 29]. An example is the recently published ultrasound study that showed the association between perineal complex imaging alterations and vaginal delivery [7].

In the future, advances in imaging for assessing perineal complex disruption are expected to enhance injury detection and enable comparative trials on the effectiveness of repair techniques, much as endoanal ultrasound research has improved the management of anal sphincter lacerations [30].

### Strengths and Limitations

Our study investigates perineal complex involvement in the prolapse mechanism by quantifying the association between PM morphology and PC_CT_ alterations, providing insights into an aspect of the pelvic support system that is rarely studied. Additionally, focusing on young women with prolapse allowed us to capture pelvic floor alterations while minimizing age-related changes that may be a confounding factor. Other technical strengths include the use of thin-slice MRI, which provides excellent images for assessing the anatomy of deep perineal tissues such as the PM. Structure identification was based on our first-hand anatomical experience in this region [1] and previously validated PM reconstruction technique [6].

Several limitations should be kept in mind. As a secondary analysis of existing scans, we had a small sample size, and groups were not matched exactly for age and parity, which warrant caution on group comparison. The parous women with normal support were selected in their original study based on a high likelihood of levator ani injury at the time of vaginal birth, and so findings might differ somewhat from the normal vaginal delivery population. But, if anything, our use of this cohort at a high risk for injury would lessen the magnitude of our findings, because the control group would be more likely to have significant birth changes not currently known than an average risk group. Our subjects are primarily white and so studies with different ethnic groups will be needed to understand differences among these populations. Last, we cannot exclude the possibility of bias owing to the lack of observer blinding.

## Conclusions

The PM structure was markedly altered in young women with prolapse, characterized by loss of the central PM connection and hiatal elongation and widening. Our findings suggest that disruption of the perineal complex—particularly its midline connective tissue component—might be associated with prolapse. Larger studies are needed to corroborate these results and, if confirmed, to inform the development and testing of surgical approaches that restore the midline connection and potentially mitigate prolapse development or progression.

## Data Availability

The data that support the findings of this study are not publicly available due to ethical restrictions but are available from the corresponding author upon reasonable request.
